# Pre‐treatments enhance the therapeutic effects of mesenchymal stem cells in liver diseases

**DOI:** 10.1111/jcmm.14788

**Published:** 2019-11-06

**Authors:** Chenxia Hu, Zhongwen Wu, Lanjuan Li

**Affiliations:** ^1^ Collaborative Innovation Center for the Diagnosis and Treatment of Infectious Diseases State Key Laboratory for the Diagnosis and Treatment of Infectious Diseases The First Affiliated Hospital School of Medicine Zhejiang University Hangzhou China; ^2^ National Clinical Research Center for Infectious Diseases The First Affiliated Hospital School of Medicine Zhejiang University Hangzhou China

**Keywords:** gene modification, liver disease, mesenchymal stem cell, pre‐treatment, survival

## Abstract

Liver diseases caused by viral infection, alcohol abuse and metabolic disorders can progress to end‐stage liver failure, liver cirrhosis and liver cancer, which are a growing cause of death worldwide. Although liver transplantation and hepatocyte transplantation are useful strategies to promote liver regeneration, they are limited by scarce sources of organs and hepatocytes. Mesenchymal stem cells (MSCs) restore liver injury after hepatogenic differentiation and exert immunomodulatory, anti‐inflammatory, antifibrotic, antioxidative stress and antiapoptotic effects on liver cells in vivo. After isolation and culture in vitro, MSCs are faced with nutrient and oxygen deprivation, and external growth factors maintain MSC capacities for further applications. In addition, MSCs are placed in a harsh microenvironment, and anoikis and inflammation after transplantation in vivo significantly decrease their regenerative capacity. Pre‐treatment with chemical agents, hypoxia, an inflammatory microenvironment and gene modification can protect MSCs against injury, and pre‐treated MSCs show improved hepatogenic differentiation, homing capacity, survival and paracrine effects in vitro and in vivo in regard to attenuating liver injury. In this review, we mainly focus on pre‐treatments and the underlying mechanisms for improving the therapeutic effects of MSCs in various liver diseases. Thus, we provide evidence for the development of MSC‐based cell therapy to prevent acute or chronic liver injury. Mesenchymal stem cells have potential as a therapeutic to prolong the survival of patients with end‐stage liver diseases in the near future.

## 1 INTRODUCTION

**Figure 1:**
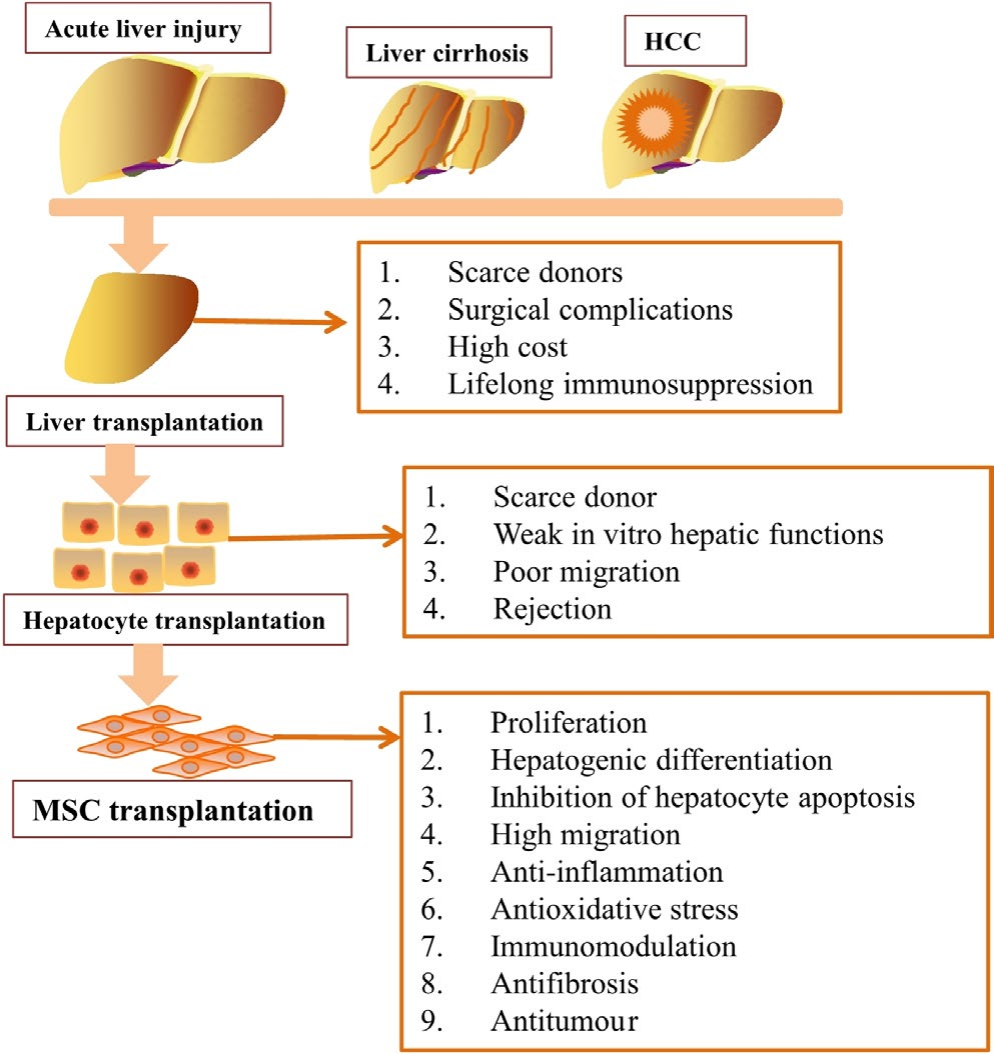
Multiple therapies, such as LT, hepatocyte transplantation and MSC transplantation, have been developed to treat patients with end‐stage liver diseases

Liver diseases induced by viral infection (eg hepatitis B and hepatitis C), autoimmune hepatitis, alcohol abuse, non‐alcoholic fatty liver disease, non‐alcoholic steatohepatitis and metabolic disorders progress to end‐stage liver failure, liver cirrhosis and liver cancer,^1^, ^2^ which are a growing cause of death worldwide.^3^ Multiple therapies have been developed to treat patients with end‐stage liver diseases by promoting liver regeneration and attenuating liver injury (Figure 1). Liver transplantation (LT) is the only effective treatment with potential long‐lasting benefits in patients with end‐stage liver diseases, but the application of LT is inhibited by a shortage of donors, surgical complications, high cost and the need for lifelong immunosuppression.^4^ Primary hepatocyte transplantation has replaced LT since transplanted hepatocytes play a critical role in liver regeneration and can compensate for impaired liver functions in vivo. Liver transplantation is a complex surgery and carries the risks of complications inherent to surgery. Although hepatocyte transplantation is less invasive and less expensive than LT, the sources of primary hepatocytes are scarce, and these cells have weak in vitro hepatic function; for these reasons, the application of these cells for treating liver diseases is limited.^5^ Moreover, only 0.1%‐0.3% of primary hepatocytes migrate into host liver tissue, leading to poor therapeutic effects in vivo.^6^ Dhawan et al^7^ demonstrated that patients who underwent hepatocyte transplantation experienced allogeneic rejection and a decline in liver graft function within 1 year. Stem cell‐based therapy has emerged as an alternative strategy to hepatocyte transplantation for improving liver function and promoting liver regeneration.

Mesenchymal stem cells (MSCs) are fibroblast‐like, adherent, immunomodulatory and multipotent cells that rapidly proliferate in vitro under specific conditions.^8^ They can be isolated from various tissues, including bone marrow, umbilical cord blood and adipose tissue, and can undergo hepatogenic differentiation upon culture in hepatic medium.^9^ MSCs restore liver function after hepatogenic differentiation and exert immunomodulatory, anti‐inflammatory, antifibrotic, antioxidative stress and antiapoptotic effects in liver cells.^10^ Although MSCs exert antitumour effects via inhibition of the Wnt signalling pathway, it is worth considering that MSCs may promote tumour initiation and growth by exerting immunosuppressive and angiogenic effects in human hepatocellular carcinoma (HCC).^11^ After isolation and culture in vitro, MSCs are faced with nutrient and oxygen deprivation, and external growth factors cannot maintain MSC capacities for further applications.^12^ Although MSCs can undergo differentiation into various somatic cells under defined conditions in vitro, they rarely transform into target cells after transplantation. In addition, transplanted MSCs undergo apoptosis or senescence in response to the harsh microenvironment.^13^ An obstacle facing MSC‐based transplantation therapy is the limited number of functional stem cells available after transplantation due to the harsh microenvironment, anoikis and inflammation induced by damaged tissues or organs.^13^ The acute in vivo inflammatory response effectively promotes the recruitment of progenitor cells; however, chronic inflammation significantly inhibits the recruitment and survival of local progenitor cells and implanted MSCs.^14^ Thus, anti‐inflammatory and paracrine mechanisms are main contributors to repairing liver tissue damage and prolonging the survival of animal models with liver injury. Mesenchymal stem cells significantly up‐regulate the secretion of the anti‐inflammatory cytokine interleukin (IL)‐10 and decrease the production of tumour necrosis factor (TNF)‐α, interferon‐gamma (IFN‐γ) and IL‐12.^15^ In addition, MSC‐derived secretomes contain protein mediators such as hepatocyte growth factor (HGF), transforming growth factor (TGF)‐β3, indoleamine 2,3‐dioxygenase (IDO) and prostaglandin 2 (PGE2), which are important for anti‐inflammatory signalling and immunoregulation.^16^

**Figure 2:**
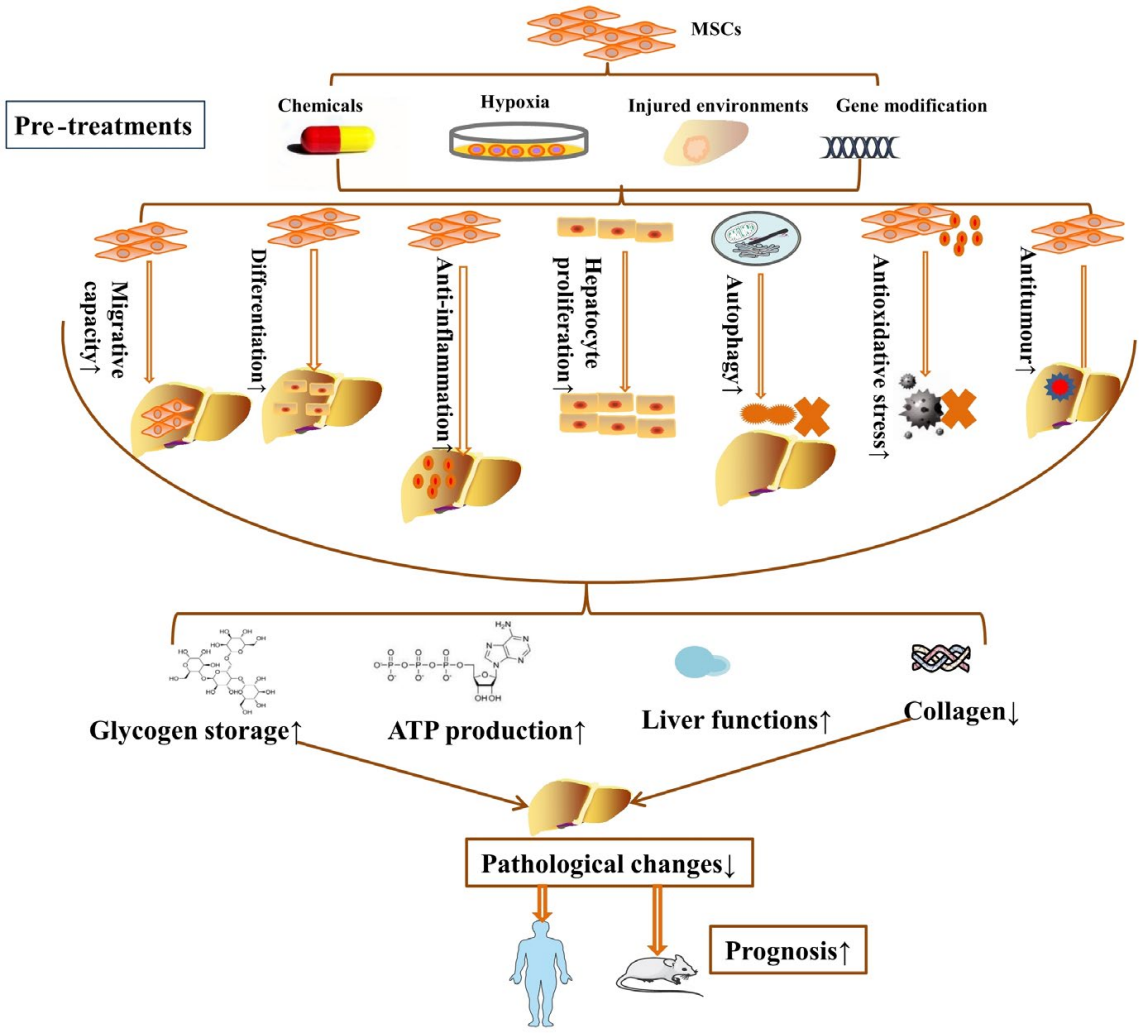
The underlying mechanisms of MSC pre‐treatments in various liver diseases

Chemical agents, hypoxia, inflammatory microenvironments and gene modification can be utilized to protect MSCs against injury induced by a harsh microenvironment, thereby improving the homing capacity, survival rate and paracrine effects of MSCs in vitro and in vivo, as well as the ability of these cells to enhance liver function.^17^, ^18^, ^19^, ^20^ In the current review, we mainly focus on pre‐treatments and the underlying mechanisms for improving the therapeutic effects of MSCs in various liver diseases (Figure 2). In this way, pre‐treated MSCs can be administered to prolong the survival of patients with end‐stage liver diseases in the near future.

## 2 THE POTENTIAL MECHANISMS BY WHICH MSC ADMINISTRATION CAN TREAT LIVER DISEASES

Mesenchymal stem cells significantly reduce inflammatory factor secretion, immune cell infiltration and hepatocyte apoptosis but up‐regulate antioxidant levels and energy metabolism in chemical‐induced acute liver injury. For example, MSCs reduced liver injury in d‐galactosamine (d‐Gal)/lipopolysaccharide (LPS)‐induced acute liver failure (ALF) rats via reducing the release of inflammatory cytokines, such as IL‐1β, IL‐6, and TNF‐α; down‐regulating the nuclear factor‐kappa B (NF‐κB) pathway; and up‐regulating the expression of haeme oxygenase‐1 (HO‐1).^21^ MSCs inhibited the infiltration of lymphocytes, dendritic cells and Kupffer cells, further decreased the serum levels of inflammatory factors (TNF‐α, interferon‐γ and IL‐4) and increased the serum levels of the hepatoprotective factor IL‐10 in mice with concanavalin A‐induced acute liver injury.^22^ Meanwhile, MSCs significantly eliminated acetaminophen‐induced injury and increased the survival rate of ALF mice via inhibiting cytochrome P450 activity and MAPK signalling but improving antioxidative activity.^23^ Traditionally, alanine aminotransferase, aspartate aminotransferase, prothrombin time, ammonia and total bilirubin have been used as biomarkers of liver injury. Mesenchymal stem cell administration was reported to improve liver function, as shown by decreased alanine transaminase and aspartate aminotransferase expression, prothrombin time and serum ammonia, via the down‐regulation of liver isoprostanes, 8‐hydroxyguanosine (8‐OHG) and nitrite nitrates and the maintenance of hepatic glutathione (GSH), which are protective factors that eliminate oxidative stress. Moreover, MSCs down‐regulated the expression levels of TNF‐α, monocyte chemoattractant protein‐1 (MCP‐1), IL‐1β, intercellular adhesion molecule 1 (ICAM‐1) and phospho‐c‐Jun NH2‐terminal kinase (p‐JNK) and increased the liver regeneration rate in acetaminophen‐induced liver injury rats.^24^ MSCs also attenuated hepatocyte apoptosis and accelerated the regeneration of remnant liver tissues in rats with major hepatectomy‐induced ischaemia reperfusion (I/R) injury.^25^ It is possible that MSCs employ redox signalling to coordinate self‐renewal and differentiation or to regulate stem cell activity in response to oxidative stress. Thus, the metabolic balance is an important regulator of MSC‐based regenerative medicine. In addition to inhibiting hepatocyte apoptosis, MSCs participated in maintaining metabolic balance by regulating amino acids, bile acids, sphingolipids, acylcarnitines and glycerophospholipids in liver cells to attenuate liver injury in ALF rats.^26^ Liver transplantation always has a high rejection rate, and although liver tissue is a tolerogenic organ with adaptive systems, acute graft‐vs‐host disease is a serious and life‐threatening complication of LT.^27^ More recently, MSC transplantation has been recognized as a novel treatment for preventing graft rejection and treating autoimmune diseases such as graft‐vs‐host disease via immunomodulatory effects mediated by cell‐to‐cell interactions and secreted cytokines.^28^ MSCs improved the prognosis of LT animals by suppressing hepatocyte apoptosis, KC apoptosis, Th1/Th17 infiltration, chemokine release and inflammatory cell infiltration.^29^ In addition, transplanted MSCs inhibited allograft rejection and activated CD4+CD25+Foxp3+ Tregs to prolong the survival of LT rats.^30^

Liver cirrhosis is a continuous liver injury in which quiescent hepatic stellate cells (HSCs) transform into proliferative, α‐smooth muscle actin + myofibroblast‐like cells that deposit collagen in liver tissue. Hepatic stellate cell activation, extracellular matrix and collagen deposition and immune cell accumulation in liver tissue result in liver fibrosis or cirrhosis in mammals.^31^ MSCs effectively induced the apoptosis of HSCs and inhibited liver inflammation and collagen deposition to block hepatic fibrosis.^32^, ^33^ Patients with hepatitis C virus‐induced liver fibrosis also benefited from MSC transplantation, which prompted the down‐regulation of fibrotic markers and inflammatory factors and the up‐regulation of anti‐inflammatory factors in liver tissue.^34^ On the other hand, MSCs improved the prognosis of patients with hepatitis B virus‐induced liver fibrosis by up‐regulating Tregs and down‐regulating Th17 cells. Subsequently, MSCs increased serum TGF‐β levels while decreasing the expression levels of IL‐17, TNF‐α and IL‐6 in these fibrotic patients.^35^

MSC administration significantly suppressed chemically induced HCC by inhibiting the Wnt and NF‐kB signalling pathways.^36^, ^37^ In contrast, MSCs were shown to promote tumour growth and metastasis in HCC patients by supporting angiogenesis and modulating the immune response in vivo.^38^ MSCs also contribute to the acceleration of HCC metastasis via the induction of epithelial‐mesenchymal transition (EMT), which further contributes to shortening the overall survival of HCC patients.^39^

## 3 TRANSPLANTED HLCS WITH IMPROVED LIVER FUNCTION MAY EFFECTIVELY PARTICIPATE IN REPAIRING THE INJURED LIVER

**Figure 3:**
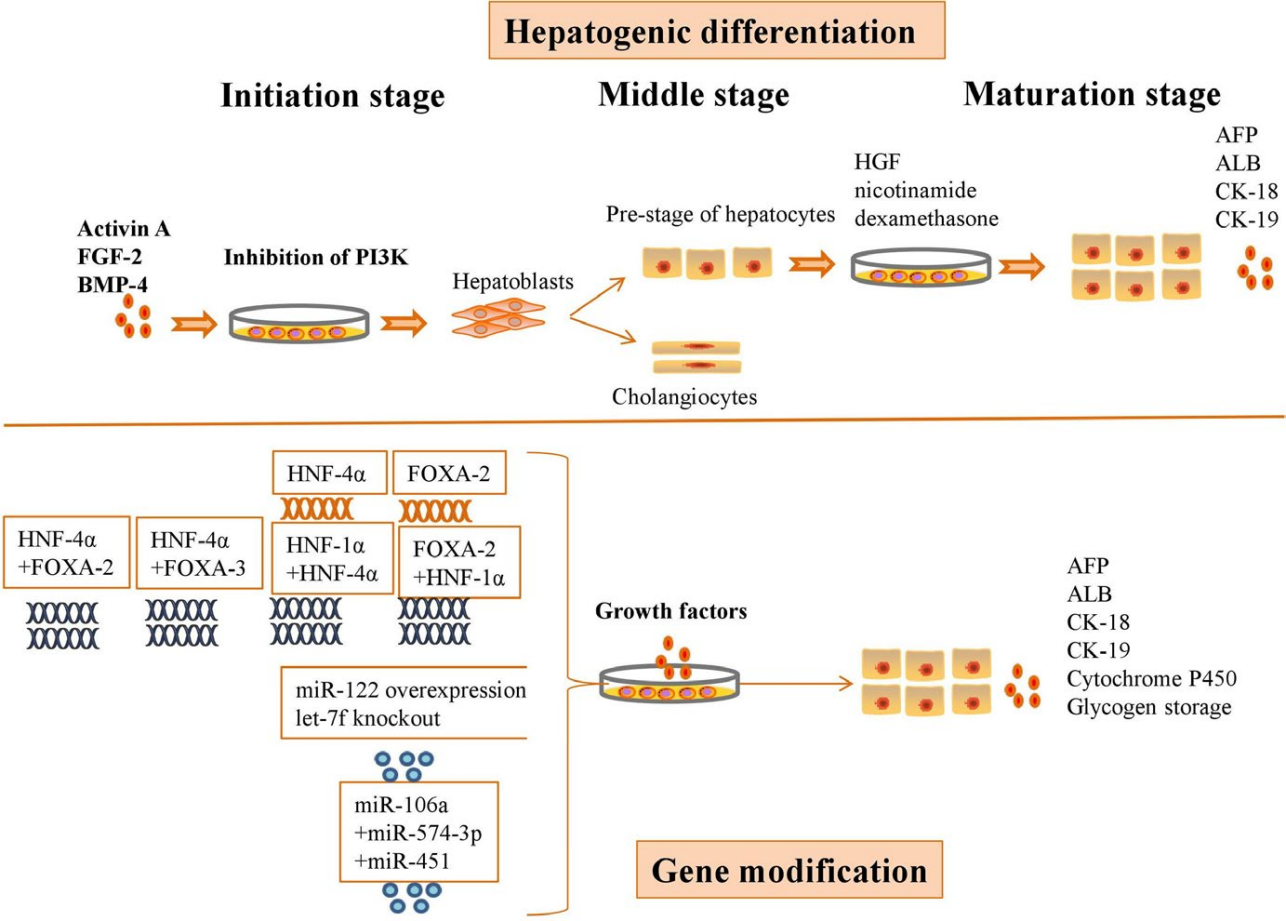
Multiple strategies by which growth factor addition or gene modification contribute to improving the liver‐specific functions of MSCs

According to current studies, MSCs can transform into hepatocytes with liver‐specific functions, but the immature phenotypes of these differentiated cells inhibit their application. Thus, multiple strategies based on the addition of growth factors or gene modification have contributed to improvements in liver‐specific functions (Figure 3).

The hepatogenic differentiation of MSCs comprises three phases: initiation, differentiation and maturation. Upon culture with activin A, fibroblast growth factor (FGF)‐2, bone morphogenetic protein (BMP)‐4 and a phosphoinositide 3‐kinase (PI3K) inhibitor, MSCs initiated hepatogenic differentiation. These multipotent cells subsequently generated multiple hepatoblasts, which can further differentiate into cholangiocytes and hepatocytes during the early differentiation stage. At the final stage, the defined growth factors promoted the generation of hepatocyte‐like cells (HLCs) with high expression levels of alpha‐fetoprotein (AFP), albumin (ALB), cytokeratin 18 (CK‐18) and cytokeratin 19 (CK‐19).^40^ MSC‐derived HLCs express high levels of hepatic genes and have liver‐specific metabolic activities after culture in medium containing HGF, nicotinamide and dexamethasone.^41^

Hepatocyte nuclear factor (HNF)‐4 plays a critical role in hepatogenic differentiation and can regulate liver regeneration. Overexpression of HNF‐4α significantly increased the expression levels of hepatic‐specific genes, liver‐enriched transcription factors and cytochrome P450 genes in hepatogenic MSCs in vitro,^19^ thus transforming these MSCs into highly functional hepatocytes via activation of the Wnt/β‐catenin pathway.^42^ Forkhead box A2 (FOXA‐2) is reported to activate liver‐specific genes, including ALB and transthyretin, to promote the hepatic differentiation of MSCs. Overexpression of HNF‐4α and FOXA‐2 promoted the maturation of MSC‐derived HLCs and up‐regulated their expression levels of ALB, urea and glucose, as well as indocyanine green uptake and cytochrome P450 activity. In addition, the transplantation of MSC‐derived HLCs was deemed safe because the transplanted cells did not form tumours after 2 months.^43^ However, basic research cannot determine the safety of MSC‐derived HLC transplantation; pre‐clinical and clinical trials are necessary. In addition, HNF‐4α and forkhead box A3 (FOXA‐3)‐overexpressing MSCs showed typical hepatocyte features, with high expression of liver‐specific markers, increased glycogen storage and indocyanine green absorption, and the cytoplasmic accumulation of neutral triglycerides and lipids.^44^ The binding of HNF‐1α and HNF‐4α in MSCs is critical for efficient hepatic expansion and maturation, as HNF‐1α is a target gene of HNF‐4α. Overexpression of FOXA‐2 and HNF‐1α promoted the hepatogenic differentiation of MSCs and improved the liver functions of HLCs, as shown by increased glycogen storage, indocyanine green absorption and lipid accumulation.^45^

miRNAs are short non‐coding RNAs that participate in regulating the expression of a large number of all mammalian protein‐encoding genes. After primary miRNAs with cap structures and poly A tails are efficiently transcribed by RNA polymerase II, the microprocessor complex Drosha cleaves primary miRNAs into hairpin precursor miRNAs of 60‐70 nucleotides in the nucleus, and another RNase, Dicer, cleaves precursor miRNAs into double‐strand mature miRNAs in the cytosol.^46^ A single strand of the siRNA or miRNA duplex is incorporated into a ribonucleoprotein effector complex known as the RNA‐induced silencing complex. This complex identifies target messages based on complementarity between the guide RNA and the mRNA, resulting in either endonucleolytic cleavage of target mRNA or translational repression.^47^ Overexpression of miR‐122 enhanced the differentiation of MSCs towards functional HLCs in the absence of other growth factors in the culture medium, and this increased differentiation was accompanied by up‐regulated levels of ALB, AFP, CK‐18, CK‐19 and HNF‐4α and improvements in urea and ALB production and glycogen deposition.^48^ The highly redundant let‐7 miRNA family contains 10 subfamilies distributed across 13 loci in mice and humans. Inhibition of let‐7b significantly promoted the generation of functional hepatocytes from MSCs via the up‐regulation of liver‐enriched transcription factors (LETFs), such as HNF‐4α and HNF‐6, and of miR‐122.^49^ Overexpression of miR‐122 and knockout of let‐7f increased the hepatogenic differentiation of MSCs by up‐regulating the expression of liver‐specific markers such as ALB, AFP, CK‐18, CK‐19 and HNF‐4α. In addition, these HLCs secreted high levels of urea and ALB and showed considerable glycogen storage capacity.^50^ Although overexpression of miR‐106a, miR‐574‐3p or miR‐451 was not able to induce the hepatogenic differentiation of MSCs, concurrent ectopic overexpression of these three miRs significantly promoted the transformation of MSCs into mature hepatocytes with hepatic morphology and ALB secretion ability.^51^

In recent years, the administration of HLCs has also contributed to the improvement of liver function in various liver diseases. Hepatocyte‐like cell transplantation significantly improved liver function in ALF mice via the secretion of TGF‐β1, IL‐6, and IL‐10.^52^ In animal models of partial hepatectomy‐induced ALF, HLC transplantation significantly down‐regulated the excessive accumulation of lipids and maintained liver function, thereby improving hepatocyte survival, inhibiting hepatocyte apoptosis and ultimately prolonging the survival of these animals.^53^ HLC transplantation also significantly attenuated liver fibrosis via up‐regulating the expression levels of HGF and Bcl‐2 in liver tissue and decreasing the levels of serum fibronectin and hepatic AFP.^54^ Although HLCs are effective at repairing liver injury in various diseases, Hu et al^31^, ^55^ reported that undifferentiated MSCs can exert greater benefits than HLCs in liver diseases because HLCs are more sensitive to harsh in vitro and in vivo environments.

## 4 PRE‐TREATMENTS IMPROVE THE THERAPEUTIC EFFECTS OF MSCS IN LIVER DISEASES

### 4.1 Chemical pre‐treatments for MSC‐based therapies for liver diseases

Rapamycin significantly enhanced the migration and anti‐inflammatory effects of MSCs via the up‐regulation of autophagy and CXCR‐4 expression without altering cell viability. Moreover, rapamycin‐pre‐treated MSCs significantly decreased the hepatic pathological changes in I/R mice induced by occluding intrahepatic blood flow for 90 minutes with an atraumatic vascular clamp; these MSCs showed enhanced migration via the CXCR‐4/CXCL‐12 axis.^56^ Pre‐treatment with anaesthetics such as dexmedetomidine and midazolam enhanced the efficacy of MSCs by increasing migratory capacity, cytokine secretion (HGF, FGF, vascular endothelial growth factor [VEGF] and insulin‐like growth factor 1 [IGF‐1]) and NF‐κB p65 nuclear translocation to protect LO2 cells from hypoxia‐reoxygenation–induced injury.^20^ Heat‐shock pre‐treatment (HSP) significantly attenuated hydrogen peroxide (H_2_O_2_)‐induced apoptosis by down‐regulating Bax and cytochrome C levels and up‐regulating Bcl‐2 levels and autophagy in MSCs. Consequently, the transplantation of MSCs exposed to HSP into I/R rats decreased serum aminotransferase levels and Suzuki scores while improving histopathology and hepatocyte proliferation.^57^

Melatonin‐pre‐treated MSCs showed increased homing capacity to the injured liver site and significantly improved the percentage of glycogen storage while decreasing collagen and lipid accumulation in fibrotic liver tissue; this outcome stemmed from decreased expression of TGF‐β1 and Bax and increased expression of matrix metalloproteinases (MMPs) and Bcl‐2.^17^ Furthermore, melatonin pre‐conditioning significantly increased the engraftment of MSCs and attenuated liver fibrosis in rat models.^58^ Pre‐treatment with 10 ng/µL SDF‐1α improved the homing rate of MSCs in vitro and in vivo, and intraperitoneal injection of resveratrol into rats with common bile duct ligation‐induced liver cirrhosis further attenuated the common pathological changes by up‐regulating sirtuin 1, CXCR‐4 and MMP‐9 and down‐regulating p53 in the liver.^59^

Although the above studies tried to improve the therapeutic effects of MSCs via multiple pathways, further studies should expand the chemical entities used to regulate MSCs for the treatment of various liver diseases. In addition, all published data focus on the effects of MSCs on I/R injury and liver fibrosis in mammals, and it is necessary to investigate more chemicals for other liver diseases, such as ALF and liver cancer.

### 4.2 Hypoxic pre‐treatment for MSC‐based therapies for liver diseases

As the oxygen concentration seen by tissue‐resident MSCs is lower than 5%, in vitro culture under hypoxic conditions may effectively mimic the in vivo microenvironment and contribute to the maintenance of MSC proliferation, differentiation, metabolic balance and other physiological processes.^18^ Moreover, hypoxic pre‐treatment significantly reduced cellular injury and promoted the proliferation of MSCs by maintaining energy metabolism in vitro.^60^ Hypoxia significantly improved the activity and migrative capacity of MSCs after activation of the AKT signalling pathway and up‐regulation of c‐Met expression. Mesenchymal stem cells cultured under hypoxic conditions showed an increased migration rate and improved therapeutic effects.^18^ Incubation under hypoxia (1% O_2_) for 24 hours increased the release of various growth factors, such as VEGF, FGF‐2, IGF‐1 and HGF, and activated signalling pathways, including the NF‐κB, JNK and extracellular signal‐regulated kinase (ERK) signalling pathways, to protect against injury in vitro.^61^ Furthermore, hypoxia (1% O_2_) pre‐treatment effectively up‐regulated the expression of CX3CR‐1 and CXCR‐4 and increased the homing rate of MSCs in SDF‐1α‐expressing ischaemic tissues.^62^ On the other hand, hypoxia (1% O_2_)‐pre‐treated MSCs markedly increased hepatocyte proliferation by up‐regulating VEGF in rats that underwent massive hepatectomy compared to normoxia‐preconditioned MSCs.^63^ It is worth noting that oxygen concentration will influence the stemness of MSCs in vitro and in vivo, and Hu et al^10^ reported that hypoxic pre‐treatments have varied from 0.5% to less than 5%. Although current studies have used approximately 1% O_2_ as hypoxic pre‐treatment for MSCs applied to treat liver diseases, further studies should compare the effects of different oxygen concentrations on MSCs to improve their therapeutic effects. Although hypoxic MSCs are widely used in regenerative medicine to repair multiple injured tissues, it is necessary to expand the investigation of hypoxic MSCs to other liver diseases, such as ALF, liver fibrosis and HCC.

### 4.3 Pre‐treatments with an altered microenvironment for MSC‐based therapies for liver diseases

Intriguingly, Waterman et al^64^ showed that MSCs transformed into a pro‐inflammatory phenotype in quiescent microenvironments but into an anti‐inflammatory phenotype in an inflammatory microenvironment. To mimic the in vivo microenvironment, various strategies in addition to hypoxia have been employed to enhance stemness and hepatogenic differentiation. Pre‐conditioning with injured liver tissue increased the expression of ALB, CK‐8, CK‐18, TAT and HNF‐1α in MSCs. These pre‐treated MSCs showed enhanced homing and differentiation abilities in liver fibrosis animal models after the up‐regulation of CK‐8, CK‐18, ALB and Bcl‐xL levels and the down‐regulation of HGF, Bax and caspase‐3 levels. Thus, these pre‐treated MSCs showed improved therapeutic effects in liver fibrosis models.^65^ Pre‐treatment with serum from carbon tetrachloride (CCl_4_)‐injured rats increased the hepatogenic differentiation of MSCs, accompanied by the up‐regulated expression of liver‐specific markers such as AFP, ALB, CK‐8 and CK‐19. The transplantation of HLCs significantly reduced liver fibrosis and improved liver function in rats with CCl_4_‐induced liver fibrosis.^66^ In addition, pre‐treatment with inflammatory factors also contributes to improving MSC‐based therapeutic effects. Although IFN‐γ and a multiple cytokine cocktail consisting of IFN‐γ, TGF‐β and retinoic acid had no effect on the immunomodulation of MSCs in vivo, they significantly enhanced the capacity of MSCs to inhibit the proliferation of CD4+ T cells and CD8+ T cells and the production of IFN‐γ.^67^ Further studies should expand the related pre‐treatments to include inflammatory factors to improve MSC‐based regenerative medicine for liver diseases because MSCs respond to inflammatory factors and alter their immunoregulatory capacities in vitro and in vivo.

### 4.4 Gene modification improves the therapeutic effects of MSCs in liver diseases

A range of genes with defined biological functions have been introduced into MSCs via viral or non‐viral vectors to improve stemness, differentiation, immunoregulation, homing capacity and other repair‐related abilities in vitro and in vivo. Overexpression of FOXA‐2 increased the expression of liver‐specific genes such as AFP, CK‐18, and ALB and increased glycogen storage and cytochrome P450 activity in MSCs. Transplantation of these MSCs in a scaffold system significantly attenuated liver injury without stem cell homing to the injured liver site in a thioacetamide (TAA)‐induced model.^68^ FOXA‐2‐overexpressing MSCs promoted the recovery of fibrotic liver tissue by enhancing MSC hepatogenic differentiation and up‐regulating the expression levels of liver‐specific genes such as AFP, CK‐18, HNF‐1α and HGF.^69^

HGF‐overexpressing MSCs protected against liver injury in ALF mouse models and prolonged their survival by increasing GSH and maintaining redox homoeostasis. Moreover, overexpression of HGF enhanced the homing rate of MSCs to injured areas and down‐regulated the Bax/Bcl‐2 ratio in liver tissues of ALF mice.^70^ HGF‐overexpressing MSCs up‐regulated the expression levels of HNF‐4α, ALB and CK‐18 to attenuate liver cirrhosis, as demonstrated by decreased levels of aminotransferases and total bilirubin.^71^ HGF‐overexpressing MSCs also decreased the levels of tissue inhibitor of metalloprotease‐1 (TIMP‐1) and the fibrogenic cytokines platelet‐derived growth factor beta polypeptide b (PDGF‐bb) and TGF‐β1 but increased the expression of MMP‐9, MMP‐13, MMP‐14 and urokinase‐type plasminogen activator in fibrotic liver tissues. Thus, HGF‐secreting MSCs obviously reduced liver fibrosis in rats after decreasing collagen deposition and improving liver function.^72^

c‐Met belongs to the transmembrane tyrosine kinase receptor superfamily and participates in the phosphorylation of multiple cellular entities.^73^ Activation of the HGF/c‐Met signalling pathway improved the engraftment of MSCs into the injured liver and contributed to liver injury repair and liver regeneration by enhancing the hepatogenic differentiation of MSCs.^74^ c‐Met‐overexpressing MSCs significantly improved the survival and liver function of ALF rats, as these cells showed enhanced homing to the injured liver.^75^ Forkhead box P3 (Foxp‐3)‐overexpressing MSCs significantly inhibited the differentiation of CD4+ T cells into splenocytes in a contact‐dependent manner, while Foxp3‐overexpressing MSCs transformed CD4+ T cells into Tregs and generated donor‐specific liver allograft tolerance.^76^

Overexpression of target genes in MSCs will induce insertional mutagenesis and epigenetic alterations; thus, we should examine these obstacles in future studies. Gene‐modified MSCs have potential for application in the treatment of various liver diseases.

## 5 CONCLUSIONS

MSC transplantation significantly attenuates acute or chronic liver injury via anti‐inflammatory and immunoregulatory effects, antioxidative stress and effects on hepatogenic differentiation in injured liver tissues, thus improving the prognosis of animal models with end‐stage liver diseases. In particular, MSCs not only attenuate liver injury but also ameliorate the degree of fibrosis in mammals with chronic diseases. At the cellular level, inflammatory signals promote the proliferation of MSCs and mesenchymal‐to‐epithelial transformation, suppress HSC differentiation into fibrogenic myofibroblasts and encourage immune cells to adopt an anti‐inflammatory phenotype. Various pre‐treatments were proven to enhance the therapeutic effects of MSCs in acute liver injury and liver fibrosis (Table 1), but no studies have clarified the effects and mechanisms of MSCs on animal models and patients with HCC. This may be attributed to the potential effects of MSCs on promoting tumour growth after administration in vivo. Multiple pre‐treatments with defined biological functions enhance the therapeutic benefits of MSCs in liver diseases, but the potential mechanisms of these pre‐treated MSCs have been only partially explained by current investigations. Although genetic modification by overexpression or gene knockout was demonstrated to improve the therapeutic efficacy of MSCs in liver diseases, the use of techniques involving viral or non‐viral modification prompts safety concerns, particularly that the modified MSCs may promote the tumorigenesis of parenchymal or mesenchymal cells. Thus, it is necessary to guarantee the safety of modified MSCs for potential clinical applications in regenerative medicine.

**Table 1:** Pre‐treated MSCs from various tissues effectively protect against liver injury and improve the prognosis of patients with liver diseases

MSC source	MSC source	Dose	Pre‐treatment	Model	Injury model	Effect	Mechanism	Ref.
Rat	Bone marrow	3 × 10^6^	Melatonin	Rat	CCl_4_‐induced liver fibrosis	Improve glycogen storage; decrease the accumulation of collagen and lipids in liver tissue	Decrease the expression of TGF‐β1 and Bax; increase the expression of MMPs and Bcl2	17
Human	Umbilical cord	1 × 10^6^	Rapamycin	Mouse	I/R	Decrease pathological changes in the liver	Enhance the migration and anti‐inflammatory effects of MSCs; up‐regulate autophagy and CXCR4 expression	56
Rat	Bone marrow	1 × 10^6^	HSP	Rat	I/R	Decrease serum aminotransferase levels and Suzuki scores; improve histopathology and hepatocyte proliferation	Attenuate H_2_O_2_‐induced apoptosis; down‐regulate Bax and cytochrome C levels; up‐regulate Bcl‐2 levels and autophagy	57
Rat	Bone marrow	1.5 × 10^6^	Melatonin	Rat	CCl_4_‐induced liver fibrosis	Attenuate liver fibrosis	Increase the engraftment of MSCs	58
Rat	Bone marrow	1 × 10^6^	Resveratrol and SDF‐1α	Rat	Common bile duct ligation‐induced liver fibrosis	Attenuate the pathological changes of liver cirrhosis	Up‐regulate the expression of sirtuin 1, CXCR4 and MMP‐9; down‐regulate p53 expression	59
Rat	Bone marrow	5 × 10^6^	Hypoxia	Rat	85% hepatectomy	Improve ALB levels, the liver weight/body weight ratio and rat survival	Up‐regulate the expression of VEGF	63
Mice	Bone marrow	1 × 10^6^	Injured liver tissue	Mouse	CCl_4_‐induced liver fibrosis	Decrease liver fibrosis; improve liver function	Up‐regulate the expression of CK8, CK18, ALB and Bcl‐xl; down‐regulate the expression of HGF, Bax and Caspase‐3	65
Rat	Adipose	1.5 × 10^6^	Serum isolated from rats with acute CCl_4_ injury	Rat	Liver fibrosis	Reduce liver fibrosis; improve liver function	Increase the hepatogenic differentiation of MSCs	66
Human	Adipose	N/A	Overexpression of FOXA2	Mouse	TAA‐induced injury	Decrease the necrotic area	Increase the levels of HGF, bFGF, VEGF‐A, IL‐10, IL‐4, IL‐6, and IL‐13	68
Rat	Bone marrow	N/A	Overexpression of FOXA2	Rat	Liver fibrosis	Promote the recovery of fibrotic liver tissue	Enhance MSC hepatogenic differentiation	69
Human	Umbilical Cord	1 × 10^6^	Overexpression of HGF	Mouse	Acetaminophen‐induced acute liver failure	Protect against liver injury in ALF mice; prolong the survival of ALF mice	Increase GSH levels; maintain redox homoeostasis; enhance the homing rate of MSCs	70
Rat	Bone marrow	1.0 × 10^6^	Overexpression of HGF	Rat	CCl_4_‐induced liver cirrhosis	Attenuate liver cirrhosis	Up‐regulate the expression levels of HNF‐4α, ALB and CK18	71
Human	Bone marrow	1 × 10^7^	Overexpression of HGF	Rat	Dimethylnitrosamine‐induced liver fibrosis	Reduce collagen deposition; improve liver function	Decrease the levels of TIMP‐1 and the fibrogenic cytokines PDGF‐bb and TGF‐β1; increase the expression of MMP‐9, MMP‐13, MMP‐14 and urokinase‐type plasminogen activator	72
Rat	Bone marrow	1.0 × 10^7^/kg	Overexpression of c‐Met	Rat	d‐GalN/LPS	Improve the survival rate and liver functions	Enhance the homing ability of MSCs	75

## CONFLICT OF INTEREST

The authors declare no competing financial interests.

## AUTHORS' CONTRIBUTIONS

Lanjuan Li contributed to the conception of this study. Chenxia Hu and Zhongwen Wu were responsible for the literature review. Chenxia Hu and Zhongwen Wu drafted and revised the manuscript. All authors read and approved the final manuscript.
